# Synthetic Approaches to Mono- and Bicyclic Perortho-Esters with a Central 1,2,4-Trioxane Ring as the Privileged Lead Structure in Antimalarial and Antitumor-Active Peroxides and Clarification of the Peroxide Relevance

**DOI:** 10.3390/molecules22010119

**Published:** 2017-01-11

**Authors:** Axel G. Griesbeck, Maria Bräutigam, Margarethe Kleczka, Angela Raabe

**Affiliations:** Department of Chemistry, University of Cologne, Greinstr. 4, 50939 Köln, Germany; marialisa.braeutigam@googlemail.com (M.B.); m.kleczka@uni-koeln.de (M.K.); angela@raabe.com (A.R.)

**Keywords:** artemisinin, peroxides, singlet oxygen, hydroperoxides, 1,2,4-trioxane, orthoester

## Abstract

The synthesis of 4-styryl-substituted 2,3,8-trioxabicyclo[3.3.1]nonanes, peroxides with the core structure of the bioactive 1,2,4-trioxane ring, was conducted by a multistep route starting from the aryl methyl ketones **1a**–**1c**. Condensation and reduction/oxidation delivered enals **4a**–**4c** that were coupled with ethyl acetate and reduced to the 1,3-diol substrates **6a**–**6c**. Highly diastereoselective photooxygenation delivered the hydroperoxides **7a**–**7c** and subsequent PPTS (pyridinium-*p*-toluenesulfonic acid)-catalyzed peroxyacetalization with alkyl triorthoacetates gave the cyclic peroxides **8a**–**8e**. These compounds in general show only moderate antimalarial activities. In order to extend the repertoire of cyclic peroxide structure, we aimed for the synthesis of spiro-perorthocarbonates from orthoester condensation of β-hydroxy hydroperoxide **9** but could only realize the monocyclic perorthocarbonate **10**. That the central peroxide moiety is the key structural motif in anticancer active GST (glutathione S-transferase)-inhibitors was elucidated by the synthesis of a 1,3-dioxane **15**—with a similar substitution pattern as the pharmacologically active peroxide **11**—via a singlet oxygen ene route from the homoallylic alcohol **12**.

## 1. Introduction

The 2015 Nobel Prize in physiology and medicine awarded to the Chinese scientist Youyou Tu, for her discoveries concerning a novel therapy against malaria [1,2], honored a lifelong work in structure elucidation and investigations on mechanism of action of potent antimalarial drugs from folk medicine. The central compound in this research is the unusual peroxide *artemisinin* (in Chinese translation: *qinghaosu*), a structurally complex tetracyclic sesquiterpene lactone with an endoperoxide substructure that can be isolated from the leaves of *Artemisia annua* [3,4,5,6,7,8]. Due to its very high antimalarial activities, artemisinin, its derivatives, and numerous analogs have become important as antimalarial drugs against multidrug-resistant forms of *Plasmodium falciparum* [9]. Recently, numerous reports have also described diverse antitumor activities of these compounds [10,11,12,13,14]. This high pharmacological potential combined with its synthetically challenging and chemically unusual structure have prompted synthetic chemists to design total or partial synthesis routes to this compound and derivatives thereof [15,16,17,18,19,20,21,22] as well as the design of artemisinin dimers, conjugates and dyads [23]. In addition, flow chemistry has been applied in order to produce large amounts of artemisinin involving a photochemical key step [24,25,26]. With respect to the chemical reactivity of artemisinin derivatives, an ongoing discussion concerns the correlation between biological activity and the kind of peroxide-related steps that actually interfere with parasite-infected cells [27,28,29,30]. 

**Scheme 1:**
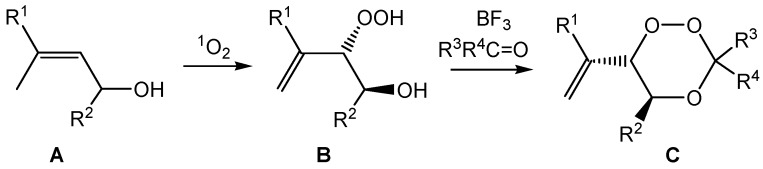
Synthetic route to 1,2,4-trioxanes using the singlet oxygen ene reaction.

The central pharmacophore of natural artemisinin and derivatives is the 1,2,4-trioxane ring. A more detailed look at the artemisinin structure shows that this group is part of a bicyclo[3.2.2]nonane skeleton, i.e., not existing in a perfect cyclohexane chair conformation. Furthermore, not only a peracetal group but also an acetal is present in artemisinin and its dihydroderivative (DHA, with a further hemiactal structure) or DHA ethers like arthemether or artheether (corresponding to the methyl and ethyl ether of DHA, respectively). A structural simplification that has been extensively realized synthetically and examined with respect to antimalaria-activity is the 3-spiroannelated 1,2,4-trioxane structure [31,32,33]. We have recently started to explore synthetic routes to these compounds as well as to ring-contracted bicyclic peroxides with intact 1,2,4-trioxane units as well as to multifunctional trioxanes [34,35,36]. Additionally, molecular dyads that combine the natural artemisinin skeleton with synthetic trioxanes were realized by our group [37,38,39]. The synthetic protocol that we follow in this approach is depicted in Scheme 1: allylic alcohols A are highly reactive substrates for the singlet oxygen ene reaction and lead to β-hydroxy hydroperoxides B with high regio- and, in the case of chiral allylic alcohols, also high *threo* diastereoselectivity. Furthermore, Broensted or Lewis acid (boron trifluoride shown as a representative example) catalyzed peroxyacetalization of B, in the presence of ketones, aldehydes (R^3^R^4^C=O) or equivalent reagents (acetals or orthoesters), did eventually result in the formation of the desired 1,2,4-trioxanes C.

**Scheme 2:**
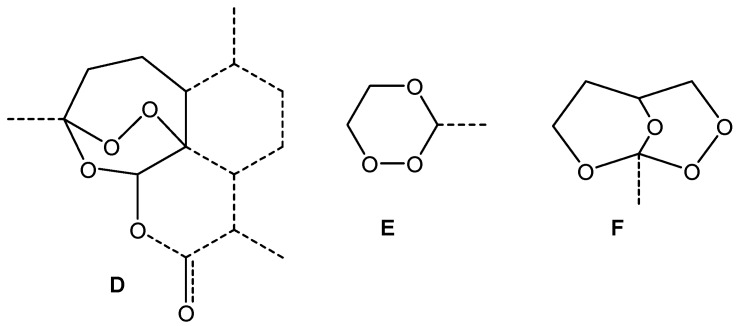
Artemisinin structure (solid lines indicating the relevant bicyclic peroxide structure), 1,2,4-trioxane and 2,3,8-bicyclo[3.3.1]nonane structures.

From chiral 3,3-dimethylated substrates (R^1^=CH_3_), diastereoisomeric mixtures of *threo* and *erythro* oxygenation products are formed in ratios that are strongly solvent-dependent [40]. Arylated substrates (R^1^=Ar) result in even higher diastereoselectivities [41,42]. We have now envisaged combining this selectivity-enhancing effect with the synthesis of multifunctional 2,3,8-trioxabicyclo[3.3.1]nonanes F, perorthoesters with the core structure of the bioactive 1,2,4-trioxane ring. From a structural point of view (Scheme 2, D = artemisinine), the combination of an additional (per)acetal group with the trioxane ring E appears to be an interesting new structural feature affecting the pharmacological properties of these compounds. 

## 2. Results and Discussion

**Scheme 3:**
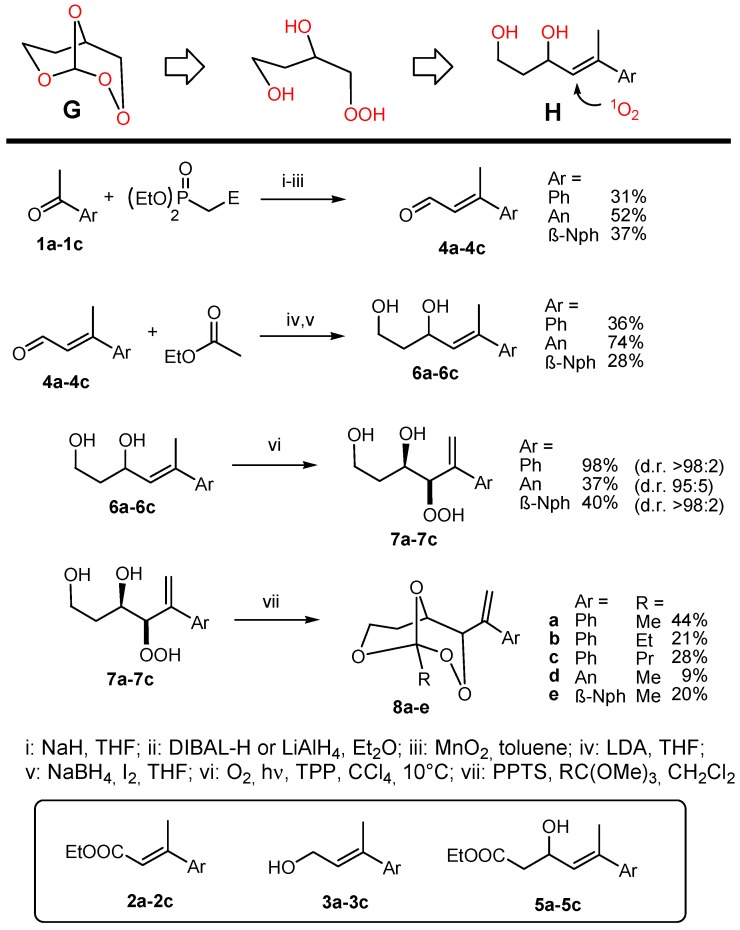
Synthesis of substrates for and products from singlet oxygen ene reactions with hydroxyethylated allylic alcohols **6**.

The synthetic protocol for the synthesis of bicyclic perorthoesters **G** as shown in Scheme 3 requires the preparation of 1-hydroperoxy-2,4-diols that are, in turn, available from allylic alcohols **H** by singlet oxygen ene reactions. The route to the 1,3-diols **6a**–**6c** starts with the Horner–Wadsworth–Emmons olefination of aric ketones **1a**–**1c** and subsequent reduction of the Michael esters **2a**–**2c** with DIBAL-H or lithium aluminum hydride. The allylic alcohols **3a**–**3c** were oxidized with MnO_2_ to give the aldehydes **4a**–**4c**. Aldol reaction with ethylacetate resulted in the aldols **5a**–**5c** that were reduced to the 1,3-diols **6a**–**6c**. The synthesis of the allylic hydroperoxides **7a**–**7c** from the allylic alcohols **6** proceeded according to literature procedures published by us for solid state photooxygenation [32,33,34].

**Scheme 4:**
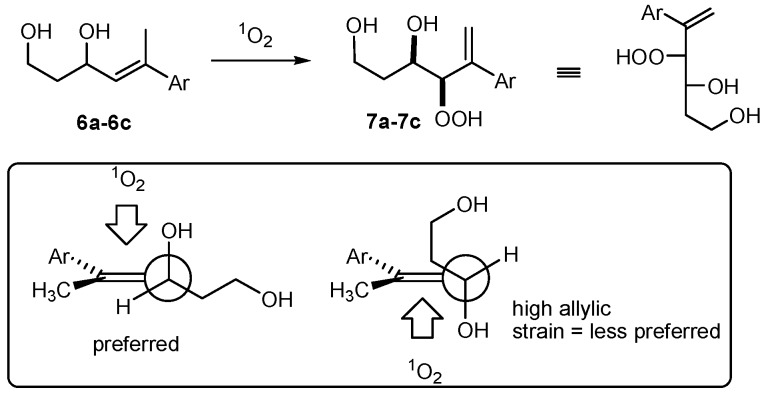
Regio- and diastereoselectivity controlling elements in the singlet oxygen ene reaction with allylic alcohols **6**.

This photooxygenation showed very high *syn* (*threo*) selectivity due to the well-known allylic hydroxy effect [40]. The selectivity was even higher (>95:5) as for the standard substrate 1,3-dimethyl-2-buten-1-ol (mesitylol, dr 85:15 under these conditions) because only the methyl group in Z-geometry to the stereogenic hydroxyl-substituted center is active in hydrogen transfer (Scheme 4). This high selectivity was also reported by Singh and coworkers for arylated (mono) allylic alcohols [41,42]. The suprafacial approach of the highly electrophilic singlet oxygen that appears from the same side as the hydroxyl group is located, indicating a hydrogen bond interaction that stabilizes the transition state.

**Scheme 5:**
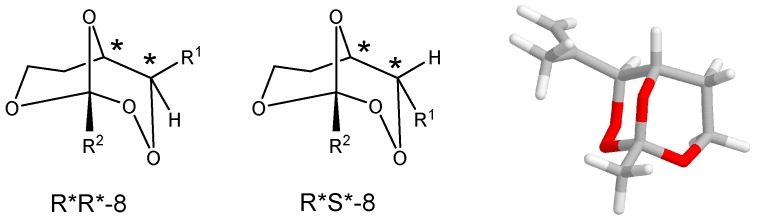
Diastereoisomeric 2,3,8-trioxabicyclo[3.3.1]nonanes (R*R*-8 preferred over R*S*-8) and force field optimized model compound without aryl substituents.

The allylic hydroperoxides **7** were suitable substrates for the final peroxyacetalization. As carbonyl reagents, orthoesters were used because three condensation steps are necessary for the formation of the bicyclic products **8**. As a Broensted acid catalyst, pyridinium-*p*-toluenesulfonic acid (PPTS) was used, and the products were obtained in moderate yields (20%–44%). Only the anisyl derivative **8d** was formed in low yields of 8%. Because of the high *threo*-diastereoselectivity of the singlet oxygen ene reaction, the products were formed as preferentially as one diastereoisomer with a pseudo-equatorial conformation of the substituent R^1^ (corresponding to the precursor with R*R*-configuration, Scheme 5). Antimalaria activities of these compounds were in the micromolar range [43]. The most active 1,2,4-trioxanes that we have obtained by analogous synthetic route showed in vitro activities against *Plasmodium falciparum* (K1 strain) in the 1–5 nM region [31], one order of magnitude higher than the perorthoesters **8**.

**Scheme 6:**
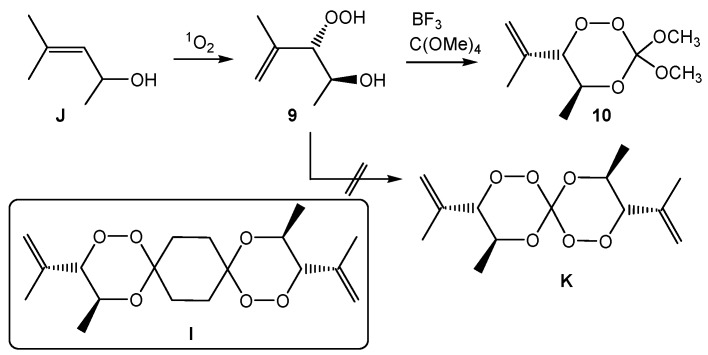
Literature-known dimeric trioxane structure **I** [36] and envisaged synthetic approach to the hexaoxaspiro[5,5]undecane structure **K**.

In order to explore the synthetic limitations of the perorthoester synthesis, the condensation of β-hydroxy hydroperoxides with orthocarbonates was investigated. As a model compound, the allylic hydroperoxide **9** (available from the singlet oxygen reaction with mesitylol **J**) was used and treated with a substoechiometric amount of tetramethylorthocarbonate. The primary peroxyacetalization proceeded smoothly to give the perorthocarbonate **10** in 30%, but no further reactivity of **10** was detected (Scheme 6).

**Scheme 7:**
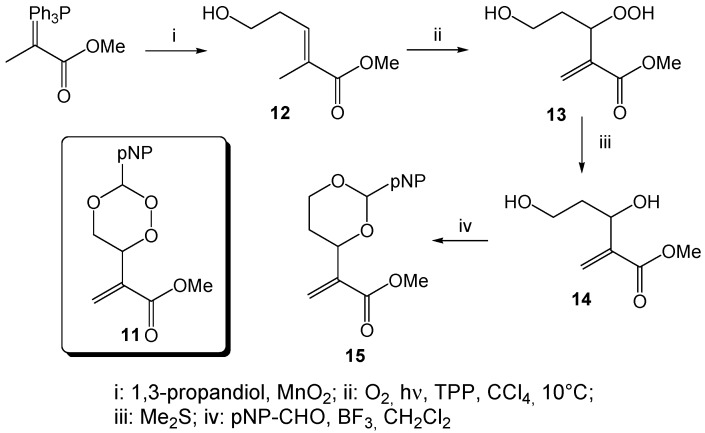
Antitumor-active 1,2,4-trioxane **11** and synthetic route to the non-peroxidic 1,3-dioxane **15**.

In recent publications, we have also demonstrated the new potential of cyclic peroxides from the 1,2,4-trioxane family to inhibit certain glutathione transferases (GSTs), an important class of detoxification enzymes that are upregulated by tumor cells [44,45]. In order to demonstrate the relevance of the central peroxide ring system, we designed the synthesis of a 1,3-dioxane **15** (Scheme 7) that is structurally related to the biologically active 1,2,4-trioxane **11** (pNP = 4-nitrophenyl) [44]. The key synthetic step was again a singlet oxygen ene reaction with the homoallylic alcohol **12**. In situ reduction of the hydroperoxide **13** with Me_2_S and acetalization delivered the dioxane **15**. No inhibition of hpGST could be determined for this compound, which accounts for the relevance of the peroxidic 1,2,4-trioxane ring in bioactive compounds.

## 3. Experimental Section

Infrared spectra were obtained using a Perkin-Elmer 1600 series FTIR spectrometer (Perkin-Elmer, Walluf, Germany) and are given in cm^−1^ units. Solid samples are measured as CsI or KBr discs while liquids are measured as neat between two NaCl plates; the ^1^H-NMR spectra were recorded on Bruker Avance 300, Bruker DPX 300 spectrometers (Bruker, Ettlingen, Germany) operating at 300 MHz, or on Bruker Avance 500 spectrometer (Bruker, Ettlingen, Germany) instruments operating at 500 MHz. Chemical shifts are reported as δ in ppm and the coupling constants *J* in Hz units. In all spectra, the solvent peaks were used as the internal standard. Solvents used are CDCl_3_ (δ = 7.24 ppm), DMSO-*d*_6_ (δ = 2.49 ppm), acetone-*d*_6_ (δ = 2.04 ppm), and MeOH-*d*_4_ (δ = 3.35, 4.78 ppm). Splitting patterns are designated as follows: s, singlet; d, doublet; t, triplet; q, quartet; m, multiplet; br, broad; the ^13^C-NMR spectra were recorded either on a Bruker Avance 300 spectrometer instrument operating at 75 MHz or on a Bruker Avance 500 spectrometer instrument operating at 125 MHz; high resolution mass spectra (HR-MS) were recorded on a Finnigan MAT 900 spectrometer(Scientific Instrument Services, Ringoes, NJ, USA) and are measured for the molecular ion peak (M^+^); CHN-combustion analyses were measured using an Elementar Vario EL Instrument (Elementar, Langenselbold, Germany).

### General Procedures (GP)

GP-1: *Horner–Wadsworth–Emmons reaction*. In a flame-dried 200 mL three-necked flask in a nitrogen atmosphere, sodium hydride (60% dispersion in oil) was washed with hexane and suspended in THF. Triethylphosphonoacetate was dropped to the suspension at 0 °C over 10 min and stirred at r.t. for 30 min. After that, a solution of the ketone in THF was added in 10 min and the reaction mixture heated to reflux for 10 h. After cooling to r.t., an aqueous NH_4_Cl solution was added and extracted with 4× diethylether. The organic phases were washed with brine and dried over Na_2_SO_4_. After solvent evaporation, the product was purified by column chromatography.

GP-2: *Reduction with DIBAL-H (diisobutylaluminium hydride)*. A solution of DIBAL-H (1.5 M in toluene) was slowly added to a solution of the ester **2** in diethylether cooled to 0 °C and subsequently stirred at r.t. for 2 h. After dilution with diethylether (twice the solvent volume of the starting reaction) and cooling to 0 °C, saturated aqueous NaCl solution was added slowly, the pH was adjusted to 3 with 4 M HCl, and the aqueous phase was extracted with 3× diethylether. The organic phases were washed with brine and dried over Na_2_SO_4_. After solvent evaporation, the product was purified by column chromatography.

GP-3: *Oxidation with MnO_2_*. A suspension of MnO_2_ in a toluene solution of the alcohol **3** was stirred overnight at RT. Afterwards, the reaction mixtures were filtered over celite and the solvent was evaporated. The crude product was purified by column chromatography.

GP-4: *Aldol reactions with ethyl acetate*. A solution of n-BuLi in hexane (19.2 mmol, 2 M) was added at −78 °C to a solution of diisopropylamine (19.2 mmol) in 40 mL of THF. After stirring for 5 min, ethyl acetate (16 mmol) was added and the mixture stirred at −78 °C for 2 h. Afterwards, the aldehyde **4** in 10 mL of THF was added and stirred for 5 min, warmed to −20 °C and quenched with 20 mL of 1 N HCl. The aqueous phase was extracted with 3× diethylether. The organic phases were washed with brine and dried over Na_2_SO_4_. After solvent evaporation, the product was purified by column chromatography.

GP-5: *Reduction of aldols with NaBH_4_*. In a flame-dried 200 mL three-necked flask in a nitrogen atmosphere, the aldol was dissolved in THF, cooled to 0 °C and NaBH_4_ was added in one portion. Afterwards, a solution of iodine in THF was slowly added and the reaction mixture heated to reflux for 3 h. After cooling to RT, methanol was carefully added until a clear solution resulted, stirred for 30 min and the solvent evaporated. The colorless residue was dissolved in a 1:1 water-diethylether solvent mixture, stirred for 20 min, separated and the aqueous phase extracted with 3× diethylether. The organic phases were washed with brine and dried over Na_2_SO_4_. After solvent evaporation, the product was purified by column chromatography.

GP-6a: *Photooxygenation under homogeneous conditions*. The substrate (1 mmol) was dissolved in 30 mL of CCl_4_ and 3–5 mg (2–4 × 10^−4^ M) of the sensitizer TPP (meso-tetraphenylporphyrin) were added. The solution was cooled to 10 °C and irradiated with a white LED under oxygen atmosphere until complete conversion (TLC = thin layer chromatography control). Subsequently, the solvent was evaporated and the residue analyzed by NMR. The crude hydroperoxides were directly used for peroxyacetalization.

GP-6b: *Photooxygenation under polymer matrix conditions*. Commercially available polystyrene-divinylbenzene copolymer beads (2.5 g) are distributed over a Petri dish (19 cm diameter) and swollen by CH_2_Cl_2_ (20 mL). The substrate (ca. 10 mmol) and the nonpolar sensitizer (TPP or TTP, ca. 3–6 mg) in ethyl acetate (20 mL) are subsequently added and the excess solvent is evaporated by leaving the Petri dish in a well ventilated hood. The Petri dish is then covered with a glass plate and the sandy solid is irradiated with a halogen lamp or a sodium street lamp. The polymer beads are subsequently rinsed with ethanol (3 × 30 mL) and filtered (the beads are kept for regeneration and reuse). The solvent is evaporated under reduced pressure (caution: water bath temperature should not exceed 30 °C).

GP-7a: *Peroxyacetalization to give bicyclic perorthoesters*. To a stirred solution of the β-hydroxy hydroperoxide **7** (1 mmol) and 3 equivalents of an orthoester in dry CH_2_Cl_2_ (12 mL) was added at room temperature a catalytic amount of pyridinium-*p*-toluenesulfonic acid (PPTS) (ca. 10 mg) and the mixture was further stirred for about 12 h (overnight) at the same temperature. The reaction mixture was partitioned between CH_2_Cl_2_ and saturated NaHCO_3_ solution and the phases were separated. The aqueous phase was extracted with CH_2_Cl_2_ (3 × 30 mL) and the combined organic phases were washed with brine and water, and dried over Na_2_SO_4_. Solvent evaporation (caution: water bath temperature should not exceed 30 °C) followed by chromatographic purification afforded the bicyclic perorthoesters as pure products.

GP-7b: *Peroxyacetalization to give monocyclic 1,2,4-trioxanes*. To a stirred solution of the β-hydroxy hydroperoxide and 1.5 equivalents of the carbonyl component in dry CH_2_Cl_2_ (100 mL) was added at room temperature a catalytic amount of boron trifluoride etherate (ca. 0.2 mL) and the mixture was further stirred for about 12 h (overnight) at the same temperature. The reaction mixture was partitioned between CH_2_Cl_2_ and saturated NaHCO_3_ solution and the phases were separated. The aqueous phase was extracted with CH_2_Cl_2_ (3 × 30 mL) and the combined organic phases were washed with brine and water, and dried over Na_2_SO_4_. Solvent evaporation (caution: water bath temperature should not exceed 30 °C), followed by chromatographic purification, afforded the spiro-bistrioxanes as pure products.

*(E)-Ethyl-3-phenylbut-2-enoate* (**2a**) [46]. Following GP-1 (General Procedure 1), 1.61 g (13.4 mmol) of acetophenone (**1a**) and 6.86 g (34.6 mmol, 2.6 eq) of triethylphosphono acetate and 1.33 g (33.4 mmol, 2.5 eq) of sodium hydride in 40 mL THF were reacted. The product was purified by column chromatography (CH/Et_2_O = 9:1, *R*_f_ = 0.22) and isolated as yellow oil: 2.0 g, 10.5 mmol, 78%. ^1^H-NMR (300 MHz, CDCl_3_): δ (ppm) = 1.32 (t, 3H, *J* = 7.1 Hz, CH_3_CH_2_), 2.59 (d, 3H, *J* = 1.2 Hz, CH_3_CH=), 4.22 (q, 2H, *J* = 7.1 Hz, CH_2_CH_3_), 6.15 (d, 1H, *J* = 1.2 Hz, CH_3_CH=), 7.35–7.49 (m, 5H, H_ar._). ^13^C-NMR (75 MHz, CDCl_3_): δ (ppm) = 14.3 (CH_3_CH_2_), 17.9 (CH_3_), 59.8 (CH_3_CH_2_), 117.1 (HC=C), 126.2 (C_ar._), 128.4 (C_ar._), 128.9 (C_ar._), 142.2 (C_ar._), 155.4 (CH_3_C=), 166.8 (C=O).

*(E)-3-Phenylbut-2-enol* (**3a**) [47]. Following GP-2 (General Procedure 2), 335 mg (1.76 mmol) of ester **2a** was reduced with 3.52 mL DIBAL-H (3.52 mmol, 2 eq, 1H solution in hexane) in 16 mL of diethylether. The product **3a** was purified by column chromatography (PE/Et_2_O 9:1, *R*_f_ = 0.12) and isolated as colorless oil: 170 mg, 1.23 mmol, 70%. ^1^H-NMR (300 MHz, CDCl_3_): δ (ppm) = 2.09 (s, 3H, CH_3_), 2.70 (br s, 1H, OH), 4.38 (d, 2H, *J* = 6.63 Hz, CH_2_OH), 6.01 (dt, 1H, *J* = 1.33 Hz, 6.63 Hz, HC=C), 7.33–7.46 (m, 5H, H_ar._). ^13^C-NMR (75 MHz, CDCl_3_): δ (ppm) = 15.8 (CH_3_), 59.8 (CH_2_OH), 125.6 (C_ar._), 126.5 (HC=C), 127.0 (C_ar._), 128.1 (C_ar._), 137.3 (CH_3_C=) 142.8 (C_ar._).

*(E)-3-Phenylbut-2-enal* (**4a**) [48]. Following GP-3 (General Procedure 3), 1.50 g (10.1 mmol) of the alcohol **3a** and 15 g of MnO_2_ in 250 mL of toluene were reacted. The product was purified by column chromatography (PE/Et_2_O = 1:1, *R*_f_ = 0.30) and isolated as colorless oil: 0.84 g, 5.7 mmol, 57%. ^1^H-NMR (300 MHz, CDCl_3_): δ (ppm) = 2.55 (s, 3H, *J* = 1.2 Hz, CH_3_), 6.39 (dq, 1H, *J* = 1.2 Hz, 7.8 Hz, HC=C), 7.35–7.55 (m, 5H, H_ar._), 10.17 (d, 1H, *J* = 7.8 Hz, CHO). ^13^C-NMR (75 MHz, CDCl_3_): δ (ppm) = 16.2 (CH_3_), 126.1 (C_ar._), 127.1 (C_ar._), 128.2 (C_ar._), 128.6 (C_ar._), 129.9 (HC=C), 140.4 (C_ar._), 157.4 (CH_3_C=), 191.0 (CHO).

*(E)-Ethyl-3-hydroxy-5-phenylhex-4-enoate* (**5a**). Following GP-4 (General Procedure 4), 840 mg (5.7 mmol) of the aldehyde **4a** and 0.3 mL (3.8 mmol) of ethyl acetate were reacted. The product **5a** was purified by column chromatography (CH/Et_2_O 3:2, *R*_f_ = 0.35) and isolated as yellow oil: 604 mg, 2.6 mmol, 68%. ^1^H-NMR (300 MHz, CDCl_3_): δ (ppm) = 1.30 (t, 3H, *J* = 7.2 Hz, CH_3_CH_2_), 2.13 (d, 3H, *J* = 1.5 Hz, CH_3_), 2.38 (m, 2H, CH_2_), 3.18 (br, 1H, OH), 4.20 (q, 2H, *J* = 7.2 Hz, CH_2_CH_3_), 5.00 (m, 1H, CHOH), 5.82 (dd, 1H, *J* = 7.2 Hz, 1.2 Hz, C=CH), 7.34–7.41 (m, 5H, H_ar._). ^13^C-NMR (75 MHz, CDCl_3_): δ (ppm) = 14.0 (CH_3_CH_2_), 16.2 (CH_3_), 41.5 (CH_2_), 60.3 (CH_2_CH_3_), 65.4 (CHOH), 125.7 (HC=C), 127.3 (C_ar._), 128.1 (C_ar._), 128.4 (C_ar._), 137.7 (C=CH), 142.6 (C_ar._), 172.1 (C=O). IR (Film): ν (cm^−1^) = 3402 (s), 2979 (m), 2928 (m), 1730 (s), 1646 (w), 1492 (w), 1369 (s), 1277 (s), 1157 (s), 1020 (s), 948 (m), 860 (m). MS (EI, 70 eV): *m*/*z* (%) = 216 (M^+^ − H_2_O, 7), 171 (4), 143 (74), 128 (100), 115 (53).

*(E)-5-Phenylhex-4-ene-1,3-diol* (**6a**). Following GP-5 (General Procedure 5), 600 mg (2.56 mmol) of the aldol **5a** was reduced with 240 mg (6.4 mmol, 2.5 eq) of NaBH_4_ and 200 mg (0.78 mmol, 0.3 eq) of I_2_. The product was purified by column chromatography (Et_2_O, *R*_f_ = 0.45) and isolated as yellow solid: 396 mg, 2.1 mmol, 80%. ^1^H-NMR (300 MHz, CDCl_3_): δ (ppm) = 1.84 (m, 2H, CH_2_), 2.10 (s, 3H, CH_3_), 2.57 (br s, 2H, OH), 3.88 (m, 1H, CH_2_OH), 4.83 (m, 1H, CHOH), 5.00 (m, 1H, CHOH), 5.84 (dd, 1H, *J* = 1.2 Hz, 8.4 Hz, C=CH), 7.30–7.40 (m, 5H, H_ar._). ^13^C-NMR (75 MHz, CDCl_3_): δ (ppm) = 15.9 (CH_3_), 38.7 (CH_2_), 60.4 (CH_2_OH), 68.1 (CHOH), 125.6 (HC=C), 127.0 (C_ar._), 128.0 (C_ar._), 130.3 (C_ar._), 136.1 (C=CH), 142.7 (C_ar._). IR (Film): ν (cm^−1^) = 3323 (s), 2943 (m), 2880 (m), 1492 (w), 1379 (s), 1121 (w), 1044 (s), 955 (m), 895 (w), 755 (s), 695 (s). MS (EI, 70 eV): *m*/*z* (%) = 177 (M^+^ − CH_3_, 9), 159 (177 − H_2_O, 4), 147 (M^+^ − OCH_2_CH_3_, 87), 129 (100), 115 (53).

*(E)-4-Methoxyethyl-3-phenylbut-2-enoate* (**2b**) [49]. Following GP-1, 2.00 g (13.4 mmol) of 4-methoxy acetophenone (**1b**) and 6.86 g (34.6 mmol, 2.6 eq) of triethylphosphono acetate and 1.33 g (33.4 mmol, 2.5 eq) of sodium hydride in 40 mL THF were reacted. The product was purified by column chromatography (CH/Et_2_O = 9:1, *R*_f_ = 0.24) and isolated as yellow oil: 2.3 g, 10.4 mmol, 77%. ^1^H-NMR (300 MHz, CDCl_3_): δ (ppm) = 1.31 (t, 3H, *J* = 7.2 Hz, CH_3_CH_2_), 2.56 (d, 3H, *J* = 1.2 Hz, CH_3_), 3.82 (s, 3H, CH_3_O), 4.20 (q, 2H, *J* = 7.2 Hz, CH_3_CH_2_), 6.11 (d, 1H, *J* = 1.2 Hz, C=CH), 6.89 (m, 2H, H_ar._), 7.45 (m, 2H, H_ar._). ^13^C-NMR (75 MHz, CDCl_3_): δ (ppm) = 14.3 (CH_3_CH_2_), 17.6 (CH_3_), 55.3 (CH_3_O), 59.6 (CH_3_CH_2_), 113.8 (C_ar._), 115.3 (CH=C), 127.6 (C_ar._), 134.3 (C_ar._), 154.8 (CH=C), 160.4 (C_ar._), 167.0 (C=O). IR (Film): ν (cm^−1^) = 2979 (s), 2914 (m), 1709 (s), 1625 (w), 1603 (m), 1513 (s), 1344 (s), 1157 (m), 1082 (m). MS (EI, 70 eV): *m*/*z* (%) = 221 (M^+^, 15), 192 (M^+^ − CH_2_CH_3_, 12), 175 (M^+^ − OCH_2_CH_3_, 100), 148 (M^+^ − O=COH_2_CH_3_, 83). CHN-analysis: (C_13_H_16_O_3_, M = 220.11 g/mol)—calcd.: 70.89% C 7.32% H, found: 71.04% C 7.63% H.

*(E)-3-(4-Methoxyphenyl)but-2-ene-1-ol* (**3b**) [50]. Following GP-2, 2.00 g (10.5 mmol) of ester **2b** was reduced with 21 mL DIBAL-H (21 mmol, 2 eq, 1 M solution in hexane) in 60 mL of diethylether. The product **3b** was purified by column chromatography (PE/Et_2_O 9:1, *R*_f_ = 0.10) and isolated as colorless oil: 1.79 g, 10.1 mmol, 96%. ^1^H-NMR (300 MHz, CDCl_3_): δ (ppm) = 2.06 (s, 3H, CH_3_), 3.81 (s, 3H, CH_3_O), 4.35 (d, 2H, *J* = 6.9 Hz, CH_2_), 5.92 (dt, 1H, *J* = 1.2 Hz, 6.9 Hz, C=CH), 6.87 (m, 2H, H_ar._), 7.36 (m, 2H, H_ar._). ^13^C-NMR (75 MHz, CDCl_3_): δ (ppm) = 16.0 (CH_3_), 55.3 (CH_3_O), 59.9 (CH_2_OH), 113.6 (C_ar._), 124.8 (C=CH), 126.8 (C_ar_.), 135.3 (CH_2_=C), 137.3 (C_ar._), 158.9 (C_ar._).

*(E)-3-(4-Methoxyphenyl)but-2-enal* (**4b**) [51]. Following GP-3, 1.79 g (10.1 mmol) of the alcohol **4a** and 18 g of MnO_2_ in 250 mL of toluene were reacted. The product was purified by column chromatography (PE/Et_2_O = 1:1, *R*_f_ = 0.28) and isolated as colorless oil: 1.79 g (6.9 mmol, 68%). ^1^H-NMR (300 MHz, CDCl_3_): δ (ppm) = 2.54 (s, 3H, CH_3_), 3.84 (s, 3H, CH_3_O), 6.38 (dd, 1H, *J* = 1.2 Hz, 7.8 Hz, C=CH), 6.93 (d, 2H, *J* = 9 Hz, H_ar._), 7.53 (d, 2H, *J* = 9 Hz, H_ar._), 10.15 (d, 1H, *J* = 7.8 Hz, CHO). ^13^C-NMR (75 MHz, CDCl_3_): δ (ppm) = 16.0 (CH_3_), 55.4 (CH_3_O), 114.0 (C_ar._), 125.5 (C=CH), 127.8 (C_ar_.), 132.4 (C_ar._), 156.9 (C=CH), 161.3 (C_ar._), 191.2 (CHO). IR (film): ν (cm^−1^) = 2957 (w), 2837 (m), 1649 (s), 1593 (s), 1506 (m), 824 (s).

(*E)-Ethyl-3-hydroxy-5-(4-methoxyphenyl)hex-4-enoate* (**5b**). Following GP-4, 325 mg (1.8 mmol) of the aldehyde **4b** and 0.13 mL (1.25 mmol) of ethyl acetate were reacted. The product **5a** was purified by column chromatography (PE/Et_2_O 1:1, *R*_f_ = 0.18) and isolated as yellow oil: 360 mg, 1.4 mmol, 77%. ^1^H-NMR (300 MHz, CDCl_3_): δ (ppm) = 1.28 (t, 3H, *J* = 7.2 Hz, CH_3_CH_2_O), 2.08 (s, 3H, CH_3_), 2.60 (m, 2H, CH_2_), 2.95 (br s, 1H, OH), 3.80 (s, 3H, CH_3_O), 4.18 (q, 2H, *J* = 7.2 Hz, CH_3_CH_2_O), 4.96 (m, 1H, CHO), 5.72 (dd, 1H, *J* = 0.9 Hz, 8.4 Hz, C=CH), 6.85 (d, 2H, *J* = 8.7 Hz, H_ar._), 7.33 (m, 2H, *J* = 8.7 Hz, H_ar._). ^13^C-NMR (75 MHz, CDCl_3_): δ (ppm) = 14.1 (CH_3_CH_2_), 16.3 (CH_3_), 41.6 (CH_2_), 55.2 (CH_3_O), 60.7 (CH_3_CH_2_), 65.8 (CHOH), 113.6 (C_ar._), 126.7 (C=CH), 126.9 (C_ar_.), 135.1 (C_ar._), 137.3 (C=CH), 159.0 (C_ar._), 172.3 (C=O). IR (Film): ν (cm^−1^) = 3429(s), 2982 (s), 2831(m), 1731 (m), 1606 (m), 1506 (s), 826 (s). MS (EI, 70 eV): *m*/*z* (%) = 264 (M^+^, 21), 249 (M^+^ − CH_3_, 12), 246 (M^+^ − H_2_O, 27), 177 (90), 159 (76), 135 (CH_3_O(C_6_H_4_)CHCH_3_, 100), 115 (39).

*(E)-5-(4-Methoxyphenyl)hex-4-ene-1,3-diol* (**6b**). Following GP-5, 175 mg (0.66 mmol) of the aldol **6a** was reduced with 63 mg (1.65 mmol, 2.5 eq) of NaBH_4_ and 51 mg (0.22 mmol, 0.3 eq) of I_2_. The product was purified by column chromatography (CHCl_3_/MeOH 9:1, *R*_f_ = 0.27) and isolated as colorless solid: 140 mg, 0.63 mmol, 96%. ^1^H-NMR (300 MHz, CDCl_3_): δ (ppm) = 1.79 (m, 2H, CH_2_), 2.04 (s, 3H, CH_3_), 3.34 (br s, 2H, 2 × OH), 3.78 (s, 3H, OCH_3_), 3.84 (m, 2H, CH_2_OH), 4.77 (m, 1H, CHOH), 5.75 (dd, 1H, *J* = 1.2 Hz, 8.4 Hz, C=CH), 6.83 (d, 2H, *J* = 8.7 Hz, H_ar._), 7.32 (d, 2H, *J* = 8.7 Hz, H_ar._). ^13^C-NMR (75 MHz, CDCl_3_): δ (ppm) = 16.0 (CH_3_), 38.8 (CH_2_), 55.1 (OCH_3_), 60.7 (CH_2_OH), 68.4 (CHOH), 113.5 (C=CH), 126.7 (C_ar._), 128.5 (C_ar._), 135.1 (C_ar._), 135.9 (C=CH), 158.8 (C_ar._). IR (Film): ν (cm^−1^) = 3332 (s), 2938 (s), 1605 (m), 1510 (s), 1245 (s), 1032 (s), 826 (m). MS (EI, 70 eV): *m*/*z* (%) = 222 (M^+^, 12), 204 (M^+^ − H_2_O, 11), 177 (C_11_H_13_O_2_^+^, 45), 159 (49), 135 (100), 115 (39).

*(E)-3-(2-Naphthyl)but-2-enylacetate* (**2c**) [52]. Following GP-1, 2.21 g (13.4 mmol) of acetonaphthone (**1c**) and 6.86 g (34.6 mmol, 2.6 eq) of triethylphosphono acetate and 1.33 g (33.4 mmol, 2.5 eq) of sodium hydride in 40 mL THF were reacted. The product was purified by column chromatography (CH/Et_2_O = 9:1, *R*_f_ = 0.39) and isolated as colorless solid, m.p. 45–46 °C, 2.5 g, 10.5 mmol, 80%. ^1^H-NMR (300 MHz, CDCl_3_): δ (ppm) = 1.36 (t, 3H, *J* = 7.2 Hz, CH_3_CH_2_), 2.71 (d, 3H, *J* = 1.2 Hz, CH_3_), 4.28 (q, 2H, *J* = 7.2 Hz, CH_3_CH_2_), 6.32 (d, 1H, *J* = 1.2 Hz, C=CH), 7.4–7.9 (m, 7H, H_ar._). ^13^C-NMR (75 MHz, CDCl_3_): δ (ppm) = 14.3 (CH_3_), 17.8 (CH_3_), 59.8 (CH_2_CH_3_), 117.4 (C=CH), 123.9 (C_ar._), 125.8 (C_ar._), 126.4 (C_ar._), 126.6 (C_ar._), 127.5 (C_ar._), 128.1 (C_ar._), 128.4 (C_ar._), 133.0 (C_ar._), 133.4 (C_ar._), 139.3 (C_ar._), 155.1 (C=), 166.8 (C=O).

*(E)-3-(2-Naphthyl)but-2-ene-1-ol* (**3c**) [53]. Following GP-2, 2.50 g (10.5 mmol) of ester **2c** was reduced with 1.11 g (29.2 mmol, 2.8 eq) of LiAlH_4_ in 16 mL of diethylether. The product **3c** was purified by recrystallization from benzene/hexane (1:1) resulting in 1.34 g (6.7 mmol, 64%) of colorless crystals, m.p. 85–86 °C. ^1^H-NMR (300 MHz, CDCl_3_): δ (ppm) = 1.67 (br s, 1H, OH), 2.20 (s, 3H, CH_3_) 4.44 (t, 2H, *J* = 5.6 Hz, CH_2_OH), 6.15 (t, 1H, *J* = 6.7 Hz, C=CH), 7.41–7.90 (m, 7H, H_ar._). ^13^C-NMR (75 MHz, CDCl_3_): δ (ppm) = 16.0 (CH_3_), 60.0 (CH_2_OH), 124.1 (C_ar._), 124.5 (C_ar._), 125.8 (C_ar._), 126.1 (C_ar._), 127.0 (C_ar._), 127.5 (C_ar._), 128.1 (C_ar._), 128.4 (C_ar._), 133.3 (C=CH), 137.6 (C_ar._), and 139.3 (C=CH).

*(E)-3-(2-Naphthyl)but-2-enal* (**4c**) [51]. Following GP-3, 1.29 g (6.5 mmol) of the alcohol **3c** and 13 g of MnO_2_ in 250 mL of toluene were reacted. The product was purified by column chromatography (PE/Et_2_O = 1:1, *R*_f_ = 0.56) and isolated as colorless oil: 0.88 g, 4.5 mmol, 69%. ^1^H-NMR (300 MHz, CDCl_3_): δ (ppm) = 2.64 (s, 3H, CH_3_), 6.55 (d, 1H, *J* = 7.8 Hz, C=CH), 7.51–8.01 (m, 7H, H_ar._), 10.23 (d, 1H, *J* = 7.8 Hz, CHO). ^13^C-NMR (75 MHz, CDCl_3_): δ (ppm) = 16.2 (CH_3_), 123.3 (C_ar._), 126.2 (C_ar._), 126.6 (C_ar._), 127.1 (C_ar._), 127.3 (C_ar._), 127.5 (C_ar._), 128.3 (C_ar._), 128.6 (C_ar._), 132.8 (C_ar._), 133.9 (C=CH), 137.4 (C_ar._), 157.1 (C=CH), 191.1 (CHO).

*(E)-Ethyl-3-hydroxy-5-(2-naphthyl)hex-4-enoate* (**5c**). Following GP-4, 875 mg (4.5 mmol) of the aldehyde **4c** and 0.30 mL (3.8 mmol) of ethyl acetate were reacted. The product was purified by column chromatography (CH/Et_2_O 3:2, *R*_f_ = 0.38) and isolated as yellow oil: 470 mg, 1.65 mmol, 37%. ^1^H-NMR (300 MHz, CDCl_3_): δ (ppm) = 1.30 (t, 3H, *J* = 7.2 Hz, CH_3_CH_2_) 2.23 (s, 3H, CH_3_), 2.66 (m, 2H, CH_2_), 4.11 (q, 2H, *J* = 7.2 Hz CH_3_CH_2_), 5.04 (m, 1H, CHOH), 5.94 (d, 1H, *J* = 7.2 Hz, C=CH), 7.45–7.82 (m, 7H, H_ar._). ^13^C-NMR (75 MHz, CDCl_3_): δ (ppm) = 14.2 (CH_3_), 16.4 (CH_2_CH_3_), 41.6 (CH_2_), 60.9 (CH_2_CH_3_), 65.7 (CHOH), 124.2 (C_ar._), 124.7 (C_ar._), 125.9 (C_ar._), 126.2 (C=CH_2_), 127.5 (C_ar._), 127.8 (C_ar._), 128.2 (C_ar._), 128.8 (C_ar._), 132.8 (C_ar._), 133.3 (C_ar._), 137.8 (C=CH_2_), 139.1 (C_ar._). IR (film): ν (cm^−1^) = 3418 (s), 3054 (m), 2979 (m), 1730 (s), 1369 (m), 1277 (m), 1158 (s), 1019 (s), 853 (m), 816 (s), 747(s). MS (EI, 70 eV): *m*/*z* (%) = 284 (M^+^, 7), 266 (M^+^ − H_2_O, 13), 196 (M^+^ − C(=O)OCH_2_CH_3_, 56), 179 (100), 155 (77), 128 (52).

*(E)-5-(2-Naphthyl)hex-4-ene-1,3-diol* (**6c**). Following GP-5, 470 mg (1.65 mmol) of the aldol **5c** was reduced with 160 mg (4.13 mmol, 2.5 eq) of NaBH_4_ and 130 mg (0.5 mmol, 0.3 eq) of I_2_. The product was purified by column chromatography (CHCl_3_/MeOH 9:1, *R*_f_ = 0.23) and isolated as colorless solid: 300 mg, 1.24 mmol, 75%. ^1^H-NMR (300 MHz, CDCl_3_): δ (ppm) = 1.89 (m, 2H, CH_2_) 2.20 (s, 3H, CH_3_), 2.29 (br s, 2H, OH), 3.94 (m, 2H, CH_2_OH), 4.90 (m, 2H, CHOH), 6.00 (dd, 1H, *J* = 1.2 Hz, 7.2 Hz, C=CH), 7.25–7.82 (m, 7H, H_ar._). ^13^C-NMR (75 MHz, CDCl_3_): δ (ppm) = 16.3 (CH_3_), 38.8 (CH_2_), 61.4 (CH_2_O), 69.2 (CHOH), 124.1 (C_ar._), 124.6 (C_ar._), 127.5 (C_ar._), 127.6 (C_ar._), 127.8 (C_ar._), 128.0 (C_ar._), 128.1 (C_ar._), 128.7 (C_ar._), 130.6 (C=CH), 133.3 (C_ar._), 136.9 (C=CH), 138.7 (C_ar._). IR (Film): ν (cm^−1^) = 3304 (s), 2921 (s), 2851 (m), 1420 (m), 1272 (m), 1046 (s), 904 (s), 854 (m), 815 (s), 727(s). MS (EI, 70 eV): *m*/*z* (%) = 242 (M^+^, 5), 192 (M^+^ − H_2_O, 5), 175 (M^+^ − OCH_2_CH_3_, 100), 148 (M^+^ − O=COH_2_CH_3_, 83). T_m_ = 38–39 °C (aus CHCl_3_).

(R*R*)-4-Hydroperoxy-5-phenylhex-5-ene-1,3-diol (**7a**). Following GP-6a, 100 mg (0.52 mmol) of the diol **6a** and 2.5 mg of TPP in 30 mL of CCl_4_ was irradiated for 5 h (complete conversion). Evaporation of the solvent delivered the crude product. ^1^H-NMR (300 MHz, CDCl_3_): δ (ppm) = 1.83 (m, 2H, CH_2_), 3.54 (m, 2H, CH_2_O), 4.42 (d, 1H, *J* = 3.9 Hz, CHOO), 4.99 (m, 1H, CHO), 5.09 (dt, 2H, *J* = 1.2 Hz, 12.6 Hz, CH_2_=C), 7.30–7.60 (m, 5H, H_ar._). ^13^C-NMR (75 MHz, CDCl_3_): δ (ppm) = 34.1 (CH_2_), 60.6 (CH_2_OH), 72.3 (CHOH), 91.3 (CHOOH), 118.0 (C=CH_2_), 126.9 (C_ar._), 128.0 (C_ar._), 128.5 (C_ar._), 139.5 (C=CH_2_), 144.9 (C_ar._).

(R*R*)-4-Hydroperoxy-5-(4-methoxyphenyl)hex-5-ene-1,3-diol (**7b**). Following GP-6a (General Procedure 6a), 126 mg (0.57 mmol) of the diol **6b** and 4 mg of TPP in 30 mL of CCl_4_ was irradiated for 5 h (37% conversion). Evaporation of the solvent delivered the crude product as a mixture of diastereoisomers (dr 95:5). ^1^H-NMR (300 MHz, CDCl_3_): δ (ppm) = 1.67 (m, 2H, CH_2_), 3.74 (s, 3H, OCH_3_), 3.88 (m, 2H, CH_2_O), 4.26 (d, 1H, *J* = 3.5 Hz, CHOO), 4.62 (m, 1H, CHO), 5.14 (d, 2H, *J* = 12.3 Hz, CH_2_=C), 6.7–7.4 (m 4H, C_ar._).

(R*R*)-4-Hydroperoxy-5-(2-naphthyl)hex-5-ene-1,3-diol (**7c**). Following GP-6b (General Procedure 6b), 200 mg (0.83 mmol) of the diol **6c** was irradiated for 5h (40% conversion). Evaporation of the solvent delivered the crude product. ^1^H-NMR (300 MHz, CDCl_3_): δ (ppm) = 1.89 (m, 2H, CH_2_) 3.97 (m, 2H, CH_2_OH), 4.34 (m, 1H, CHOO), 4.65 (m, 2H, CHOH), 5.81 (m, 2H, C=CH_2_), 7.25–7.82 (m, 7H, H_ar._). ^13^C-NMR (75 MHz, CDCl_3_): δ (ppm) = 35.8 (CH_2_), 60.2 (CH_2_O), 69.8 (CHOH), 124.1 (C_ar._), 124.6 (C_ar._), 127.5 (C_ar._), 127.6 (C_ar._), 127.8 (C_ar._), 128.0 (C_ar._), 128.1 (C_ar._), 128.7 (C_ar._), 129.4 (C=CH_2_), 133.3 (C_ar._), 138.7 (C_ar._), 139.7 (C=CH_2_).

(R*R*)-1-Methyl-4-(1-phenylvinyl)-2,3,8,9-tetraoxabicyclo[3.3.1]nonane (**8a**). Following GP-7a (General Procedure 7a), 224 mg (0.5 mmol) of the hydroperoxide **7a** was reacted with 0.08 mL (1.5 mmol, 3 eq) of trimethyl orthoacetate and catalytic amounts of PPTS in 6 mL of dichloromethane. The product was purified by column chromatography (PE/Et_2_O 9:1, *R*_f_ = 0.43) and isolated as colorless oil (dr 94:6): 57 mg (0.23 mmol, 46%). ^1^H-NMR (300 MHz, CDCl_3_): δ (ppm) = 1.50 (s, 3H, CH_3_), 1.74 (m, 1H, CH_2_), 2.45 (m, 2H, CH_2_), 3.94 (m, 1H, CH_2_O), 4.21 (d, 1H, *J* = 6.3 Hz, CHO), 4.74 (m, 1H, CH_2_O), 4.95 (s, 1H, CHOO), 5.58 (s, 1H, C=CH_2_), 5.73 (s, 1H, C=CH_2_), 7.35 (m, 5H, H_ar._). ^13^C-NMR (75 MHz, CDCl_3_): δ (ppm) = 24.1 (CH_3_), 27.5 (CH_2_), 60.0 (CH_2_O), 65.7 (CHO), 82.4 (CHOO), 113.5 (OCOO), 117.4 (C=CH_2_), 126.9 (C_ar._), 127.9 (C_ar._), 128.5 (C_ar._), 139.2 (C_ar._), 144.2 (C=CH_2_). IR (Film): ν (cm^−1^) = 2962 (m), 2923 (m), 2853 (m), 1493 (w), 1442 (m), 1380 (s), 1256 (s), 1105 (s), 966 (m), 900 (m), 752 (s), 702 (s). 

(R*R*)-1-Ethyl-4-(1-phenylvinyl)-2,3,8,9-tetraoxabicyclo[3.3.1]nonane (**8b**). Following GP-7a, 224 mg (0.5 mmol) of the hydroperoxide **7a** was reacted with 0.08 mL (1.5 mmol, 3 eq) of triethyl orthopropionate and catalytic amounts of PPTS in 6 mL of dichloromethane. The product was purified by column chromatography (PE/Et_2_O 4:1, *R*_f_ = 0.38) and isolated as colorless oil (dr 95:5): 27 mg (0.10 mmol, 21%). ^1^H-NMR (300 MHz, CDCl_3_): δ (ppm) = 1.00 (t, 3H, *J* = 7.5 Hz, CH_3_CH_2_), 1.75 (m, 3H, CH_3_CH_2_, CH_2_), 2.44 (m, 1H, CH_2_), 3.91 (m, 1H, CH_2_O), 4.21 (d, 1H, *J =* 6.6 Hz, CHO), 4.68 (m, 1H, CH_2_O), 4.96 (s, 1H, CHOO), 5.57 (s, 1H, C=CH_2_), 5.71 (s, 1H, C=CH_2_), 7.35 (m, 5H, H_ar._). ^13^C-NMR (75 MHz, CDCl_3_): δ (ppm) = 6.7 (CH_3_CH_2_), 27.8 (CH_2_), 30.8 (CH_3_CH_2_), 59.6 (CH_2_O), 65.8 (CHO), 82.9 (CHOO), 114.7 (OCOO), 117.6 (C=CH_2_), 127.0 (C_ar._), 128.0 (C_ar._), 128.5 (C_ar._), 139.1 (C_ar._), 144.4 (C=CH_2_). IR (Film): ν (cm^−1^) = 2975 (m), 1493 (w), 1360 (m), 1250 (s), 1105 (s), 914 (s), 777 (s), 702 (s).

(R*R*)-4-(1-Phenylvinyl)-1-propyl-2,3,8,9-tetraoxabicyclo[3.3.1]nonane (**8c**). Following GP-7a, 224 mg (0.5 mmol) of the hydroperoxide **7a** was reacted with 0.24 mL (1.5 mmol, 3 eq) of trimethyl orthobutyrate and catalytic amounts of PPTS in 6 mL of dichloromethane. The product was purified by column chromatography (PE/Et_2_O 4:1, *R*_f_ = 0.48) and isolated as colorless oil (dr 97:3): 39 mg (0.14 mmol, 28%). ^1^H-NMR (300 MHz, CDCl_3_): δ (ppm) = 0.92 (t, 3H, *J* = 7.5 Hz, CH_3_CH_2_), 1.54 (m, 2H, CH_3_CH_2_), 1.71 (m, 3H, CH_3_CH_2_CH_2_, CH_2_), 2.42 (m, 1H, CH_2_), 3.21 (m, 1H, CH_2_O), 4.21 (dd, 1H, *J* = 1.8 Hz, 6.6 Hz, CHO), 4.68 (m, 1H, CH_2_O), 4.96 (s, 1H, CHOO), 5.57 (s, 1H, C=CH_2_), 5.71 (s, 1H, C=CH_2_), 7.35 (m, 5H, H_ar._). ^13^C-NMR (75 MHz, CDCl_3_): δ (ppm) = 14.0 (CH_3_CH_2_), 15.7 (CH_3_CH_2_CH_2_), 27.8 (CH_2_), 39.6 (CH_3_CH_2_), 59.6 (CH_2_O), 65.6 (CHO), 82.8 (CHOO), 114.4 (OCOO), 117.5 (C=CH_2_), 126.9 (C_ar._), 127.9 (C_ar._), 128.5 (C_ar._), 139.1 (C_ar._), 144.4 (C=CH_2_). IR (Film): ν (cm^−1^) = 2963 (s), 2928 (s), 2872 (s), 1732 (m), 1493 (w), 1378 (m), 1247 (s), 1119 (s), 928 (s), 777 (s), 701 (s). 

(R*R*)-4-(1-(4-Methoxyphenyl)vinyl)-1-methyl-2,3,8,9-tetraoxabicyclo[3.3.1]nonane (**8d**). Following GP-7a, 120 mg (0.54 mmol) of the hydroperoxide **7b** was reacted with 0.21 mL (1.62 mmol, 3 eq) of triethyl orthoacetate and catalytic amounts of PPTS in 6 mL of dichloromethane. The product was purified by column chromatography (PE/Et_2_O 4:1, *R*_f_ = 0.34) and isolated as colorless oil: 14 mg (0.05 mmol, 9%). ^1^H-NMR (300 MHz, CDCl_3_): δ (ppm) = 1.39 (s, 3H, CH_3_), 1.70 (m, 1H, CH_2_), 2.45 (m, 1H, CH_2_), 3.70 (s, 3H, CH_3_O), 3.82 (m, 1H, CH_2_O), 4.09 (dt, 1H, *J* = 1.9 Hz, 6.3 Hz, CHO), 4.61 (m, 1H, CH_2_O), 4.83 (s, 1H, CHOO), 5.40 (s, 1H, C=CH_2_), 5.51 (s, 1H, C=CH_2_), 6.76 (d, 2H, *J* = 8.8, H_ar._), 7.22 (d, 2H, *J* = 8.8 Hz, H_ar._). ^13^C-NMR (75 MHz, CDCl_3_): δ (ppm) = 24.1 (CH_3_), 27.8 (CH_2_), 55.3 (CH_3_O) 59.8 (CH_2_O), 65.8 (CHO), 82.6 (CHOO), 113.6 (OCOO), 113.9 (C_ar._), 116.2 (C=CH_2_), 128.0 (C_ar._), 131.3 (C_ar._), 143.5 (C=CH_2_), 159.3 (C_ar._). IR (Film): ν (cm^−1^) = 2961 (s), 1606 (m), 1510 (s), 1464 (w), 1380 (m), 1246 (s), 1107 (m), 966 (m), 835 (s), 804 (m), 744 (w). 

(R*R*)-1-Methyl-4-(1-(2-naphthyl)vinyl)-2,3,8,9-tetraoxabicyclo[3.3.1]nonane (**8e**). Following GP-7a, 220 mg (0.8 mmol) of the hydroperoxide **7c** was reacted with 0.21 mL (1.62 mmol, 2 eq) of triethyl orthoacetate and catalytic amounts of PPTS in 8 mL of dichloromethane. The product was purified by column chromatography (PE/Et_2_O 4:1, *R*_f_ = 0.36) and isolated as colorless oil (dr 95:5): 50 mg (0.17 mmol, 20%). ^1^H-NMR (300 MHz, CDCl_3_): δ (ppm) = 1.49 (m, 1H, CH_2_), 1.75 (s, 3H, CH_3_), 2.28 (m, 1H, CH_2_), 3.91 (m, 1H, CH_2_O), 4.29 (m, 1H, CH_2_O), 4.36 (s, 1H, CHO), 5.28 (s, 1H, CHOO), 5.51 (s, 1H, C=CH_2_), 5.58 (s, 1H, C=CH_2_), 7.41–7.80 (m, 7H, H_ar._). ^13^C-NMR (75 MHz, CDCl_3_): δ (ppm) = 22.4 (CH_3_), 28.1 (CH_2_), 58.8 (CH_2_O), 76.9 (CHO), 80.0 (CHOO), 113.7 (OCOO), 120.1 (C=CH_2_), 124.7 (C_ar._), 124.8 (C_ar._), 126.2 (C_ar._), 126.4 (C_ar._), 127.6 (C_ar._), 128.0 (C_ar._), 128.3 (C_ar._), 132.9 (C_ar._), 133.2 (C_ar._), 136.1 (C_ar._), 145.8 (C=CH_2_). IR (film): ν (cm^−1^) = 3054 (m), 2966 (m), 2925 (m), 1731 (m), 1397 (s), 1300 (s), 1120 (s), 1012 (s), 942 (m), 855 (s), 750 (m). MS (EI, 20 eV): *m*/*z* (%) = 282 (M^+^ − CH_3_, 5), 266 (M^+^ − O_2_, 2), 240 (M^+^ − O_2_CCH_3_, 30), 222 (M^+^ − CH_3_CO_3_, 22), 195 (27), 184 (Naphthyl − C(CH_2_CH_3_)=CH_2_, 82), 165 (Naphthyl − C(=CH_2_)CH_3_, 57), 155 (Naphthyl − C=CH_2_, 100). 141 (Naphthyl − CH_2_, 27), 128 (Naphthyl, 27), 75 (CH_3_CO_3_, 56), 57 (CH_3_CO_2_, 89).

*3,3-Dimethoxy-5-methyl-6-(1-propen-2-yl)-1,2,4-trioxane* (**10**). Following the general procedure GP-6, 60 mg (0.45 mmol, 1.0 eq) of the hydroperoxide *threo*-**9**, 62 mg (0.03 mL, 0.23 mmol, 0.5 eq) of tetramethoxymethane and 12 mg (0.045 mmol, 0.1 eq) PPTS were reacted to give 28 mg (0.14 mmol, 30%) of the trioxane 10 after column chromatography (c-Hex/EtOAc (1:1), *R*_f_ = 0.85; ^1^H-NMR (300 MHz, CDCl_3_): δ (ppm): 1.17 (d, 3H, ^3^*J* = 6.0 Hz, O-CH-CH_3_), 1.77 (s, 3H, C-CH_3_), 3.44/3.49 (s, 2 × 3H, O-CH_3_), 4.26 (d, 1H, ^3^*J* = 8.6 Hz, C-CH-OO), 4.28–4.37 (m, 1H, CH_3_-CH-O), 4.13/4.30 (d, 1H, ^3^*J* = 8.6 Hz, CH-OOH). 5.13 (s, 2H, C=CH_2_); ^13^C-NMR (75.5 MHz, CDCl_3_): δ (ppm): 16.2 (q, 1C, O-CH-CH_3_), 19.5 (q, 1C, C-CH_3_), 51.4/52.3 (q, 2C, C-O-CH_3_), 71.0 (d, 1C, CH_3_-CH-O), 87.5 (d, 1C, C-CH-OOH), 118.7 (t, 1C, C-CH_2_), 137.0 (s, 1C, C=CH_2_). CHN-analysis: C_12_H_22_O_3_, (M = 214.30 g/mol), calcd. C 67.26% H 10.35% found: C 66.91% H 9.68%.

*Methyl-5-hydroxy-2-methylpent-2-enonate* (**12**). A mixture of 0.47 mL of propane-1,3-diol (0.5 g, 6.57 mmol) and 5.0 g of the ylene Methyl-2-(triphenylphosphoranyliden)propanoate [54] was dissolved in 17 mL of dichloromethane and 10.4 g of activated MnO_2_ were added. The mixture was stirred for 3 days at r.t. and filtered. The solid residue was washed with methylene chloride and, after solvent evaporation, purified by column chromatography (hexane/EtOAc 1:4, *R*_f_ = 0.52) to give 447 mg (3.10 mmol, 47%) **12** as a yellow oil. ^1^H-NMR (300 MHz, CDCl_3_): δ = 1.81 (s, 3H), 2.40 (q, *J* = 6.7 Hz, 2H), 3.68 (m, 5H), 6.74 (t, *J* = 7.3 Hz, 1H); ^13^C-NMR (75 MHz, CDCl_3_): δ = 12.6 (q), 32.1 (t), 51.9 (q), 61.2 (t), 129.6 (s), 138.7 (d), 168.6 (s).

*Methyl 3,5-dihydroxy-2-methylenpentanoate* (**14**). A chloroform solution of the hydroperoxide **13** (1.93 g, 10.9 mmol), obtained from the photooxygenation of the homoallylic alcohol **12** (following GP-6a), was treated with 16.5 mL of dimethylsulfide (13.9 g, 223 mmol) and stirred for 18h. After solvent and excess reagent evaporation, the residue was purified by column chromatography (CH/EtOAc 2:3, *R*_f_ = 0.30) to give 397 mg (2.25 mmol, 21%) **14** as a colorless oil. ^1^H-NMR (300 MHz, CDCl_3_): δ = 1.66 (m, 1H), 1.84 (m, 1H), 3.68 (m, 5H), 4.61 (dd, *J* = 3.1; 8.5 Hz, 1H), 5.86 (s, 1H), 6.19 (s, 1H); ^13^C-NMR (75 MHz, CDCl_3_): δ = 37.7 (t), 51.9 (q), 60.5 (t), 69.6 (d), 125.1 (t), 142.5 (s), 166.8 (s).

*Methyl-2-(2-(4-nitrophenyl)-1,3-dioxan-4-yl)acrylate* (**15**). A mixture of 711 mg of the diol **14** (4.00 mmol) and 737 mg of 4-nitrobenzaldehyde (4.80 mmol) in 38 mL of dry CH_2_Cl_2_ was treated with a stoechiometric amount (0.5 mL) of BF_3_ × Et_2_O and stirred for 18 h at r.t. After addition of 5 mL of aqueous Na_2_CO_3_, extraction with diethyl ether and solvent evaporation, the residue was purified by column chromatography (CH/EtOAc 3:1, *R*_f_ = 0.45) to give 346 mg (1.18 mmol, 49%) **15** as a yellowish solid, mp 99 °C; ^1^H-NMR (300 MHz, CDCl_3_): δ = 1.72 (dd, *J* = 12.0, 4.7 Hz, 1H), 1.86 (t, *J* = 21.2 Hz, 1H), 3.72 (s, 3H), 4.01 (t, *J* = 11.8 Hz, 1H), 4.24 (dd, *J* = 11.4, 4.5 Hz, 1H), 4.73 (d, *J* = 10.6 Hz, 1H), 5.64 (s, 1H), 5.95 (s, 1H), 6.28 (s, 1H), 7.61 (d, *J* = 8.3 Hz, 2H), 8.14 (d, *J* = 8.4 Hz, 2H); ^13^C-NMR (75 MHz, CDCl_3_): δ = 31.9 (t), 52.1 (q), 67.3 (t), 75.0 (d), 99.8 (d), 123.5 (d), 125.8 (t), 127.3 (d), 140.1 (s), 145.0 (s), 148.2 (s), 165.8 (s); IR: ν (cm^−1^) = 1031 (s), 1093 (s), 1118 (s), 1215 (s), 1273 (m), 1292 (m), 1345 (s), 1437 (m), 1520 (s), 1607 (m), 1630 (m), 1933 (w), 2858 (w), 2951 (w); CHN analysis (C_14_H_15_NO_6_): calcd. C 57.34 H 5.16 N 4.78, found C 57.08 H 5.25 N 4.67.
